# Computational design of new molecular scaffolds for medicinal chemistry, part II: generalization of analog series-based scaffolds

**DOI:** 10.4155/fsoa-2017-0102

**Published:** 2017-11-30

**Authors:** Dilyana Dimova, Dagmar Stumpfe, Jürgen Bajorath

**Affiliations:** 1 Department of Life Science Informatics, B-IT, LIMES Program Unit Chemical Biology & Medicinal Chemistry, Rheinische Friedrich-Wilhelms-Universität, Dahlmannstr. 2, D-53113 Bonn, Germany

**Keywords:** analog series, bioactive compounds, computational methods, medicinal chemistry, scaffolds, targets

## Abstract

**Aim:**

Extending and generalizing the computational concept of analog series-based (ASB) scaffolds.

**Materials & methods:**

Methodological modifications were introduced to further increase the coverage of analog series (ASs) and compounds by ASB scaffolds. From bioactive compounds, ASs were systematically extracted and second-generation ASB scaffolds isolated.

**Results:**

More than 20,000 second-generation ASB scaffolds with single or multiple substitution sites were extracted from active compounds, achieving more than 90% coverage of ASs.

**Conclusion:**

Generalization of the ASB scaffold approach has yielded a large knowledge base of scaffold-capturing compound series and target information.

## Lay abstract

‘Scaffolds’ represent core structures of small molecules. A series of structural analogs contains the same scaffold (core) and different functional groups attached to the core. Scaffolds are often used to organize active compounds into different structural families. They are also used to aid in compound design in medicinal chemistry. Scaffolds can be extracted computationally from single molecules or, following a recently introduced method, from analog series. Herein, an extension of the series-based scaffold methodology is presented to further increase the number of informative scaffolds that can be obtained and represent as many analog series and compounds as possible.

## Graphical abstract

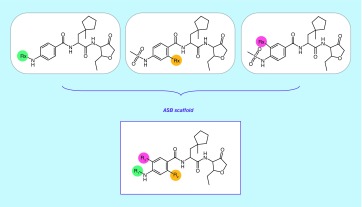

The concept of analog series-based scaffolds was further extended and taking multiple substitution sites into account. The figure shows an exemplary second-generation analog series-based scaffold.

**Key terms**
Analog series (AS): Series of related compounds sharing the same core structure and having different substituents at one or more sites.
Analog series-based (ASB) scaffold: Scaffolds derived from analog series instead of individual compounds taking chemical reaction information into account.
Matched molecular pair (MMP): A pair of compounds that are only distinguished by a chemical modification at a single site. Hence, an MMP represents two structural analogs.
Matching molecular series (MMS): Extension of the MMP definition to any series of two or more analogs that are only distinguished by a chemical modification at a single site.
Scaffold: A term describing the core structure of a compound or series.
Substitution site: Position (site) of chemical variation in a core structure carrying different substituents (functional groups, R-groups) in different analogs.

In chemistry, the scaffold concept is applied to represent core structures of small molecules [1–3]. Scaffolds are equally relevant for computational and medicinal chemistry. For example, computational analysis aims to systematically isolate and compare core structures of active compounds. In addition, computational searches for molecules with different cores having similar activity are often carried out [1,2]. In medicinal chemistry, the scaffold concept is applied to organize or design compound series, explore structure–activity relationship (SAR) information or generate focused compound libraries [3]. SAR exploration does not stringently depend on applying the scaffold concept. However, scaffolds are often generated to represent compound series and study the effects of substitutions, for example, in standard R-group tables, which continue to be the most widely used SAR data format in the practice of medicinal chemistry.

The first formal and generally applicable scaffold definition was introduced by Bemis and Murcko two decades ago [1]. The Bemis–Murcko scaffold of a compound was defined to consist of all of its ring structures and linker fragments connecting rings. Thus, a scaffold was obtained from a compound by removing all substituents (R-groups) [1]. Thereby, substitution sites were eliminated. This scaffold definition followed a molecular hierarchy by distinguishing core structures and substituents. Core structures were defined to exclusively consist of ring systems with varying topology. This scaffold definition paved the way for a consistent computational analysis of core structures and thus contributed substantially to the popularity and utility of scaffold analysis [1,3].

Recently, a different scaffold definition has been introduced to represent core structures of analog series (ASs), rather than individual compounds, and thereby further increase the utility of scaffolds for capturing core structure information from compound series [4]. So-called analog series-based (ASB) scaffolds were designed to represent unique ASs and incorporate synthetic information. However, first-generation ASB scaffolds were limited to a single substitution site to differentiate between analogs and were thus only applicable to ASs with single substitution sites.

In this work, we extend and generalize the ASB scaffold methodology by introducing second-generation ASB scaffolds that are derived from ASs with varying numbers of substitution sites. Extracting ASB scaffolds from ASs with multiple substitution sites maximizes the coverage of ASs and compounds belonging to different series and further increases the SAR information content of ASB scaffolds.

## Concepts, methods & materials

### First-generation ASB scaffolds

In the following, the two-stage ASB scaffold approach originally introduced in [4] is summarized. In stage 1, ASs are systematically extracted from compound datasets on the basis of matched molecular pairs (MMPs) [5] using a previously reported algorithm [6]. An MMP is defined as a pair of compounds that are only distinguished by a chemical modification at a single site [5]. Accordingly, an MMP consists of a shared MMP core and a pair of exchanged substituents (substructures). For ASB scaffold analysis, specialized MMPs are generated through molecular fragmentation according to retrosynthetic rules [7], yielding Retrosynthetic Combinatorial Analysis Procedure RECAP-MMPs (RMMPs) [8]. For exchanged substituents, size restrictions are introduced to limit chemical modifications to those typically observed in ASs [9].

RMMPs are then organized in a network in which nodes represent compounds and edges pairwise RMMP relationships. Each disjoint cluster in this network contains a unique AS [6]. In stage 2, all possible RMMP cores of an AS are analyzed. If a core exists that is shared by all analogs, it is defined as the ASB scaffold representing the series. Accordingly, the core is required to capture all pairwise MMP relationships within the AS. From a first-generation ASB scaffold, all analogs comprising an AS can be regenerated through chemical modifications at a single substitution site.

**Figure 1.**
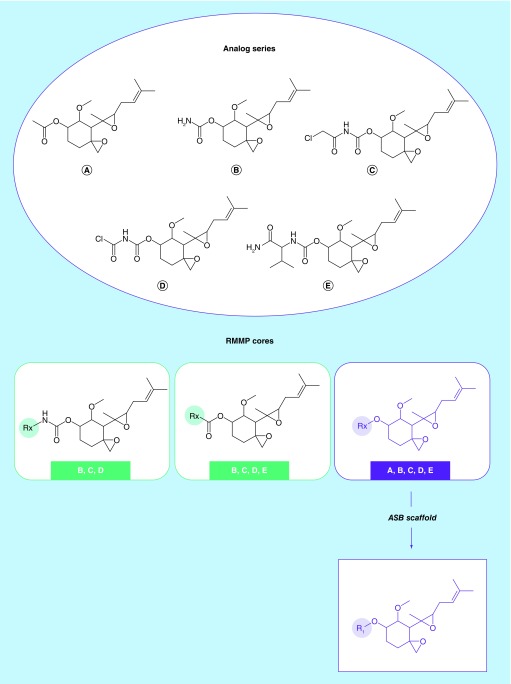
**First-generation analog series-based scaffolds.** For an AS with five analogs, all RMMP cores are shown. The core shared by all analogs A–E represents the ASB scaffold (purple) of the AS. AS: Analog series; ASB: Analog series-based scaffold; RMMP: RECAP-matched molecular pair. Adapted with permission from [4].

Figure 1 illustrates the selection of an ASB scaffold from alternative RMMP cores originating from an AS. Different from Bemis–Murcko scaffolds, which are isolated from single compounds, ASB scaffolds capture structural and activity (target) information of ASs. Importantly, a given AS yields one and only one ASB scaffold, rather than one or more Bemis–Murcko scaffolds. From a chemical perspective, an AS should contain one unique core, not multiple cores. Accordingly, ASB scaffolds are designed for AS representation consistent, for example, with the design principles of R-group tables, as discussed above.

The generation of ASB scaffolds can also be rationalized by considering an extension of the MMP concept termed matching molecular series (MMS). An MMS is defined as a series of two or more analogs that are distinguished by chemical modifications at a single site [10]. Thus, an MMS represents the union of all MMPs with shared compounds having chemical changes at the same site. Accordingly, an MMS represents an AS with a single substitution site and yields an ASB scaffold. However, as further discussed below, an AS might consist of several MMSs giving rise to the presence of multiple substitution sites within a series.

In the original study introducing ASB scaffolds [4], more than 12,000 first-generation ASB scaffolds were extracted from ChEMBL [11] (version 21), the major public repository for compounds from medicinal chemistry. In addition, in a pilot application, ASB scaffolds were successfully applied to ligand-based target deconvolution of cancer cell line screens [12].

Although a large number of ASB scaffolds were obtained, ASs and compound coverage were limited. The original method extracted ASB scaffolds from approximately 70% of all ASs in ChEMBL (version 21). The composition of the remaining 30% of the series prohibited the generation of first-generation ASB scaffolds that were confined to ASs with single substitution sites [4], as discussed above.

### Second-generation ASB scaffolds

Two methodological extensions were introduced to further increase the coverage of ASs:

- An MMP core modification operation was implemented to reduce RMMP cores with structural extensions at a given substitution site to the smallest possible core and eliminate redundant cores for a given site. Nonredundant RMMP cores represented second-generation ASB scaffolds with single substitution sites.
- One or more analogs shared by different MMSs forming an AS were identified. Then, the substitution sites of the corresponding nonredundant RMMP cores were transferred to the shared analogs, thereby accounting for multiple substitution sites. From shared analogs with mapped substitution sites, a second-generation ASB scaffold with multiple substitution sites was extracted.

### Active compounds & analog series

From ChEMBL (version 22), compounds with available high-confidence activity data were selected using a standardized data curation protocol [4]. From selected compounds, second-generation ASs were systematically extracted.

### Implementation

The extended ASB scaffold method and routines for compound selection and activity data analysis were implemented using in-house Perl and Python scripts and JAVA programs with the aid of KNIME [13] protocols and the OpenEye chemistry toolkit [14].

## Results & discussion

### Study concept & methodological extensions

The ASB scaffold approach was extended to generalize ASB scaffolds, in other words, derive them from ASs containing single as well as multiple substitution sites. Generalization of ASB scaffolds aimed to maximize ASs and compound coverage and further increase the SAR information content of ASB scaffolds.

If an AS contains a single substitution site, it represents – by definition – an MMS. The presence of multiple substitution sites in an AS results from several MMSs forming the series. In this case, each MMS must overlap with another at least once. This means that two MMSs must share at least one analog with structural modifications at two distinct sites, in other words, one substitution site per MMS, which therefore participates in two RMMPs. Accordingly, such a relationship between two MMSs gives rise to the presence of two substitution sites in the AS. Each MMS overlap in an AS then yields an additional substitution site.

To extend the ASB scaffold methodology, first, redundant RMMP cores were identified and removed, thereby reducing an AS to one or multiple nonredundant cores, depending on the number of MMSs forming the AS. Series yielding multiple qualifying RMMP cores including redundant cores were originally excluded from first-generation ASB scaffold generation.

**Figure 2.**
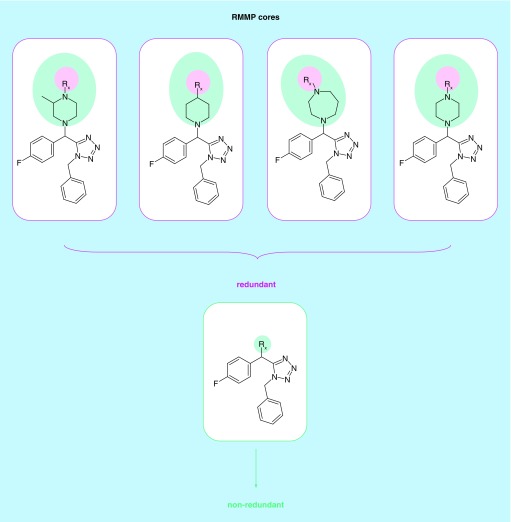

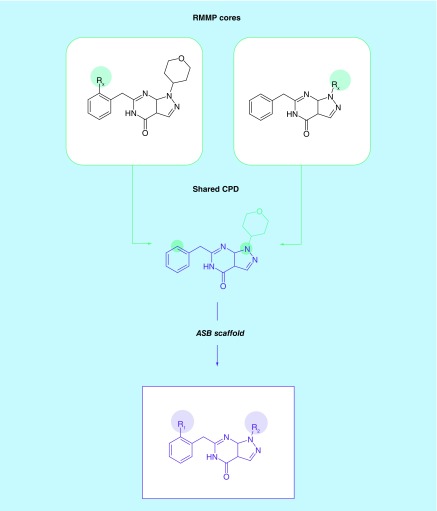

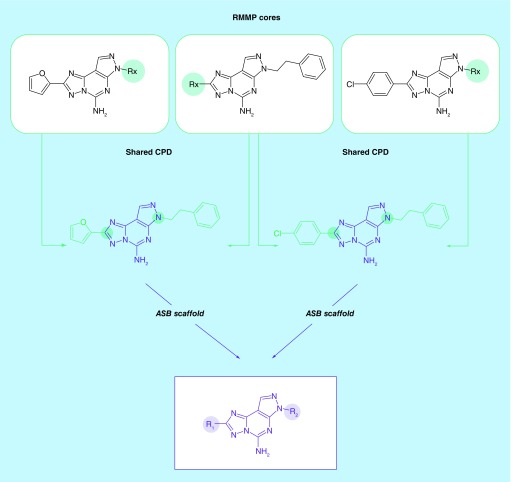

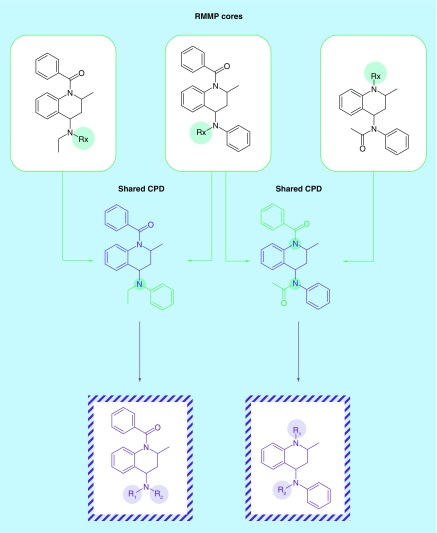
**Derivation of second-generation analog series-based scaffolds.** **(A)** The identification of redundant RMMP cores is illustrated. In **(B & C)**, the generation of analog series-based (ASB) scaffolds from ASs consisting of two and three matching molecular series (MMSs), respectively, is shown. **(D)** depicts an AS with three MMSs for which substitution sites cannot be unambiguously mapped to a single scaffold. Such MMSs are excluded from ASB scaffold generation. Substitution sites in RMMP cores (Rx), mapped attachment point atoms in shared compounds (green circles), and substitution sites in resulting ASB scaffolds (R_n_) are highlighted. ASB: Analog series-based; CPD: Compound; RMMP: RECAP-matched molecular pair.

Figure 2A illustrates how a nonredundant core is obtained from multiple RMMP cores by determining the smallest possible core variant for a given substitution site. In this example, an AS yielding five RMMP cores is shown. Four of these cores represent a core extension of the smallest (nonredundant) RMMP core. Therefore, RMMP cores with core extensions are classified as redundant and removed from the AS. All compounds comprising an AS can be generated from the nonredundant RMMP core. Hence, this core represents the ASB scaffold of the AS.

In addition, ASs consisting of multiple MMSs yield multiple nonredundant RMMP cores. In these cases, analogs shared between MMSs are identified and substitution sites are transferred from nonredundant cores to shared analogs. From shared compounds with mapped substitution sites, a second-generation ASB scaffold of a given AS is derived that captures multiple substitution sites. This is illustrated in Figure 2B & C for an AS containing two and three MMSs, respectively. It is also possible that mapped substitution sites cannot be transferred to a single scaffold. An example is shown in Figure 2D. In such cases, no ASB scaffold is obtained. However, such instances are rare.

### Analog series

From ChEMBL (version 22), a total of 22,015 ASs were extracted containing 133,441 compounds with available high-confidence activity data. ASs statistics are provided in Table 1. ASs spanned a wide size range: 57% of the ASs had single-target activity and 43% were active against multiple targets. Multi-target activity is captured by ASB scaffolds. Notably, 15,625 ASs (71%) only contained a single substitution site. This was the same proportion of ASs with a single substitution site as in the original study [4] on the basis of ChEMBL (version 21) when 17,371 ASs yielded 12,294 first-generation ASB scaffolds. However, although the majority of ASs had a single substitution site – and hence yielded a first-generation ASB scaffold – these ASs only contained 38.4% of all compounds in ASs. It follows that first-generation ASB scaffolds were predominantly obtained for ASs of limited size. Accordingly, confined compound coverage by ASs with single substitution sites provided further motivation for generalizing the ASB scaffold concept. The ASs identified herein were the source for second-generation ASB scaffolds.

**Table 1.** **Analog series.**

**Statistics**	**All ASs**	**ASs with single substitution site**		**ASs with multiple substitution site**

		**First/second**	**Second**	**Second**
Number of ASs	22,015	15,625 (71.0%)	1521 (6.9%)	3226 (14.7%)

Single target	12,603	9497	779	1615

Multiple targets	9412	6128	742	1611

Number of CPDs	133,441	51,308 (38.4%)	15,837 (11.9%)	27,913 (20.9%)

AS size (CPDs)	2–767	2–60	3–168	3–166

MEAN	6.1	3.3	10.4	8.7

MEAN substitution sites	1.3	1.0	1.0	2.1

Number of targets	1446	1251	786	957

The size and target distribution of analog series (ASs) extracted from ChEMBL (version 22) compounds (CPDs) with available high-confidence activity data are reported. Statistics are separately provided for ASs with single substitution sites from which first-generation analog series-based (ASB) scaffolds are obtained (first/second; i.e., included in second-generation ASB scaffolds) and ASs with single and multiple substitution sites that are covered in addition by second-generation ASB scaffolds (second).

### Second-generation ASB scaffolds

**Figure 3.**
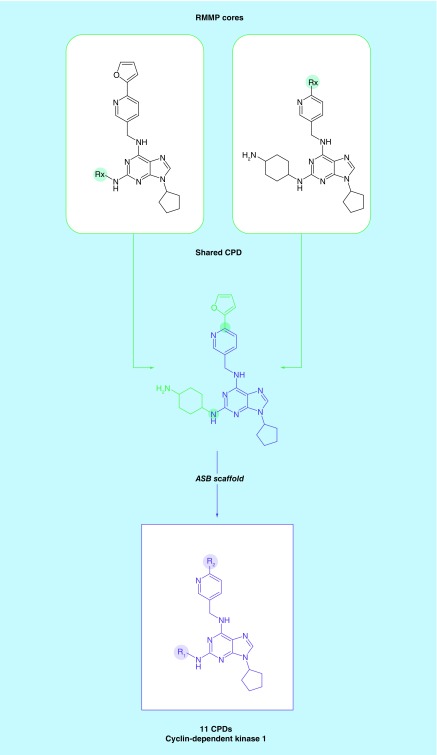

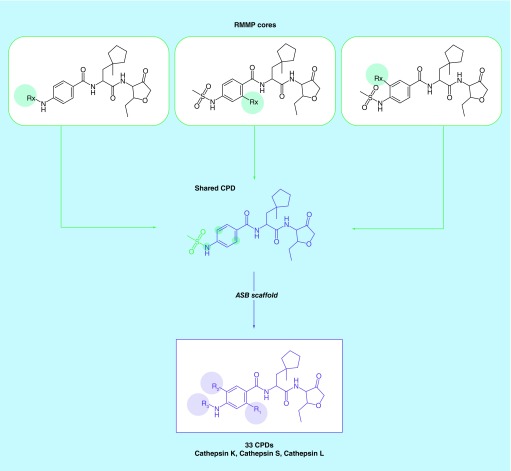
**Exemplary second-generation analog series-based scaffolds.** ASB scaffolds containing multiple substitution sites are shown derived from **(A)** 11 cyclin-dependent kinase and **(B)** 33 cathepsin K, L and M inhibitors. ASB: Analog series-based scaffold; CPD: Compound.

**Figure 4.**
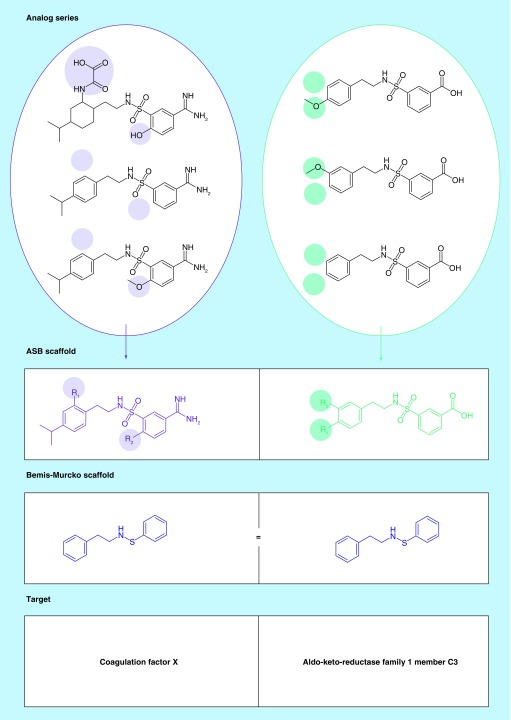

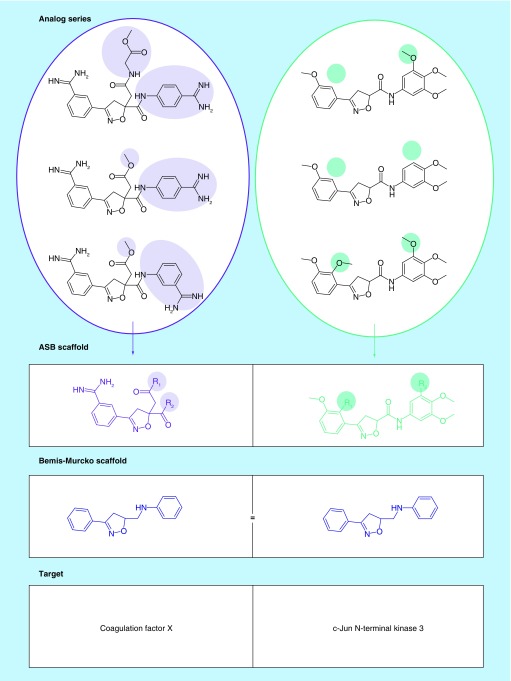

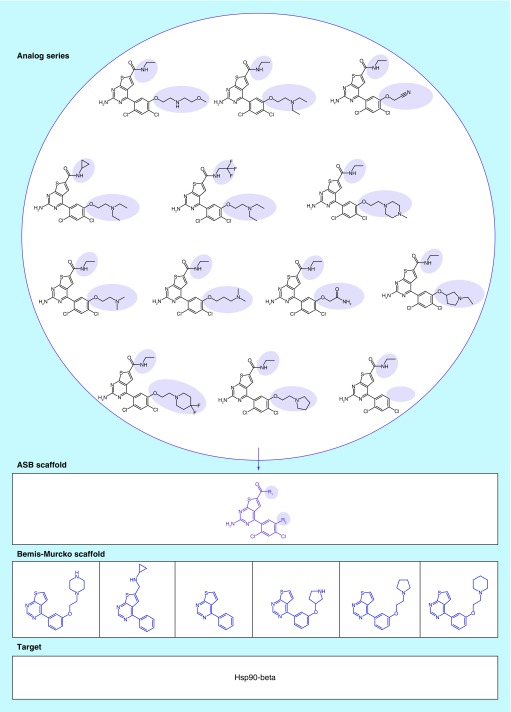

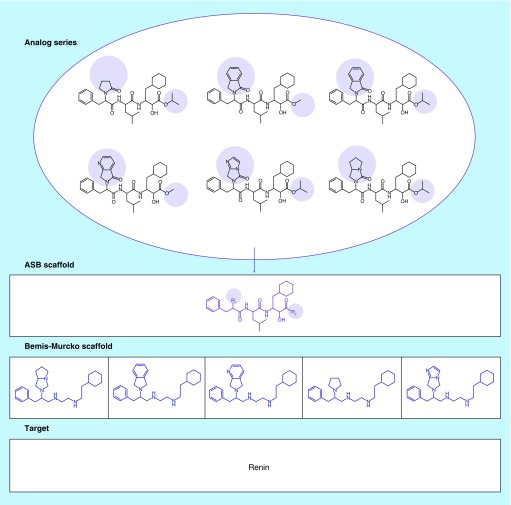
**Scaffold relationships.** In **(A & B)**, second-generation analog series-based (ASB) scaffolds from pairs of small analog series (ASs) with three analogs each are color-coded according to substitution sites. Each AS from a pair is active against a different target. The analogs share the same Bemis–Murcko scaffold (blue) but each AS yields a different ASB scaffold. In **(C & D)**, larger ASs of inhibitors of heat shock protein Hsp90-beta and renin are shown, respectively, which yield a single second-generation ASB scaffold (purple) but multiple Bemis–Murcko scaffolds (blue).

The methodological extensions detailed above made it possible to extract ASB scaffolds from ASs that were on average larger than those yielding first-generation ASB scaffolds. As reported in Table 1, the use of nonredundant RMMP cores enabled the generation of ASB scaffolds for ASs with single substitution sites that contained on average 10.4 compounds. Furthermore, ASB scaffolds were obtained from ASs with multiple substitution sites that consisted of on average 8.7 analogs. Compared with first-generation ASB scaffolds, the coverage of ASs with single substitution sites increased by 6.9% (Table 1). Moreover, extracting ASB scaffolds from ASs with multiple substitution sites further increased the coverage by 14.7%. Thus, compared with first-generation ASB scaffolds, the total ASs coverage increased from 71.0 to 92.6% and analog coverage increased from 38.4 to 71.2% (Table 1). For a small proportion of unusually large ASs with multiple substitution sites, more than one scaffold would be required to represent all analogs per series. These ASs were not considered to consistently ensure desirable one-to-one correspondence of ASs and ASB scaffolds. A total of 20,372 second-generation ASB scaffolds were obtained representing 95,058 compounds with activity against 1381 unique targets. These ASB scaffolds provide a large pool of information-rich core structure representations. Figure 3 shows exemplary second-generation ASB scaffolds with multiple substitution sites and their target annotations. In addition, Figure 4 provides examples of second-generation ASB scaffolds with further increased SAR information content compared with Bemis–Murcko scaffolds and first-generation ASB scaffolds. In Figure 4A & B, exemplary pairs of ASs are shown that yield the same Bemis–Murcko scaffold but different series-specific ASB scaffolds with multiple substitution sites. The pairs of ASs are structurally related but each AS from a pair is active against a different target. Thus, in these cases, second-generation ASB scaffolds differentiate between related ASs with different activities. In Figure 4C & D, larger ASs with activity against different targets are shown that yield single second-generation ASB, but multiple compound-based scaffolds. Hence, each ASB scaffold uniquely represent an AS, which is meaningful from a chemical and SAR perspective.

## Conclusion & future perspective

Two basic ideas provided the foundation for the development of the ASB scaffold concept. First, scaffolds representing ASs contain more SAR and target information than compound-based scaffolds. Second, an AS should contain one and only one formally defined scaffold. Compounds forming an AS might be active against single or multiple targets. This information is captured at the level of ASB scaffolds. In addition, different from compound-based scaffolds, an ASB scaffold contains the structural framework conserved within an AS. Importantly, the ASB scaffold concept does not distinguish between a core structure and substituents and thereby covers all structural elements conserved in an AS that are relevant for bioactivity. Thus, ASB scaffolds are not restricted structurally by a predefined molecular hierarchy. In addition, taking synthetic information into account further increases the utility of computationally generated scaffolds for the practice of medicinal chemistry, for example, scaffold-based compound design.

First-generation ASB scaffolds were confined to ASs having single substitution sites, which represent 71% of currently available ASs but only less than 40% of the compounds participating in ASs. Therefore, we have attempted to generalize the ASB scaffold approach by making it applicable to ASs containing multiple substitution sites. To achieve this goal, methodological extensions of the ASB scaffold approach were designed and implemented. Applying the further extended methodology, ASs and compound coverage of second-generation ASB scaffolds exceeded 90 and 70%, respectively.

Given these enhancements, the potential of ASB scaffolds for medicinal chemistry applications further increases. We also anticipate that ASB scaffolds representing analogs with single- or multitarget activity will provide interesting templates for the design of focused compound libraries. This is also supported by applying retrosynthetic rules to obtain ASB scaffolds instead of random bond fragmentation, as originally used for MMP generation.

In the near future, we intend to compute second-generation ASB scaffolds from all currently available bioactive compounds, including new database releases, and make a comprehensive collection of ASB scaffolds with target annotations freely available for drug discovery and design applications.

**Executive summary**

**Concepts, methods & materials**

- Extensions of the analog series-based (ASB) scaffold methodology are introduced to capture multiple substitution sites and maximize analog series (ASs) and compound coverage.
- Only nonredundant RECAP-matched molecular pair cores are retained and substitution sites from multiple matching molecular series forming an AS are combined.

**Results**

- More than 20,000 second-generation ASB scaffolds were extracted from compounds with available high-confidence activity data.
- Generalized ASB scaffolds approach complete ASs and compound coverage.

**Conclusion & future perspective**

- Methodological modifications are designed to generalize the ASB scaffold methodology.
- Second-generation ASB scaffolds are expected to have further increased utility for medicinal chemistry.
